# Emergent HIV-1 Drug Resistance Mutations Were Not Present at Low-Frequency at Baseline in Non-Nucleoside Reverse Transcriptase Inhibitor-Treated Subjects in the STaR Study

**DOI:** 10.3390/v7122943

**Published:** 2015-12-07

**Authors:** Danielle P. Porter, Martin Daeumer, Alexander Thielen, Silvia Chang, Ross Martin, Cal Cohen, Michael D. Miller, Kirsten L. White

**Affiliations:** 1 Gilead Sciences, 333 Lakeside Drive, Foster City, CA 94404, USA; silvia.chang@gilead.com (S.C.); ross.martin@gilead.com (R.M.); cal.cohen@gilead.com (C.C.); michael.miller@gilead.com (M.D.M.); kirsten.white@gilead.com (K.L.W.); 2 Seq-IT GmbH & Co. KG, Pfaffplatz 10, 67655 Kaiserslautern, Germany; m.daeumer@immungenetik-kl.de (M.D.); a.thielen@immungenetik-kl.de (A.T.)

**Keywords:** rilpivirine, efavirenz, resistance, virologic failure, minority variants

## Abstract

At Week 96 of the Single-Tablet Regimen (STaR) study, more treatment-naïve subjects that received rilpivirine/emtricitabine/tenofovir DF (RPV/FTC/TDF) developed resistance mutations compared to those treated with efavirenz (EFV)/FTC/TDF by population sequencing. Furthermore, more RPV/FTC/TDF-treated subjects with baseline HIV-1 RNA >100,000 copies/mL developed resistance compared to subjects with baseline HIV-1 RNA ≤100,000 copies/mL. Here, deep sequencing was utilized to assess the presence of pre-existing low-frequency variants in subjects with and without resistance development in the STaR study. Deep sequencing (Illumina MiSeq) was performed on baseline and virologic failure samples for all subjects analyzed for resistance by population sequencing during the clinical study (*n* = 33), as well as baseline samples from control subjects with virologic response (*n* = 118). Primary NRTI or NNRTI drug resistance mutations present at low frequency (≥2% to 20%) were detected in 6.6% of baseline samples by deep sequencing, all of which occurred in control subjects. Deep sequencing results were generally consistent with population sequencing but detected additional primary NNRTI and NRTI resistance mutations at virologic failure in seven samples. HIV-1 drug resistance mutations emerging while on RPV/FTC/TDF or EFV/FTC/TDF treatment were not present at low frequency at baseline in the STaR study.

## 1. Introduction

Drug resistance testing for HIV-infected patients is recommended by treatment guidelines as part of standard of care to inform the selection of initial, as well as next-line treatment strategies after virologic failure [1]. Standard genotyping assays use population sequencing, which detects mutations present in the majority of the virus quasispecies (approximately ≥20% of the virus population). Deep sequencing (or next-generation sequencing; NGS) technologies enable the detection of low-frequency (minority) quasispecies down to a threshold of ~1%–2% of the virus population. The clinical significance of low-frequency viral variants on treatment response is unclear. Some studies have shown an association between the presence of pre-existing low-frequency drug resistant variants with an increased risk of virologic failure, particularly for non-nucleoside reverse transcriptase inhibitors (NNRTIs), while other studies have shown no significant impact of low-frequency variants on virologic outcome [2,3,4,5,6,7,8,9,10,11].

The STaR study (GS-US-264-0110) was a randomized, open-label, 96-week study designed to directly compare the safety and efficacy of the two NNRTI-based single tablet regimens (STRs) rilpivirine (RPV)/emtricitabine (FTC)/tenofovir disoproxil fumarate (TDF) and efavirenz (EFV)/FTC/TDF in treatment-naïve HIV-1-infected adults [12,13]. At Week 96, resistance development to at least one component of RPV/FTC/TDF occurred more frequently than resistance development to EFV/FTC/TDF (5.3% *vs.* 1.0%, respectively). Furthermore, within the RPV/FTC/TDF arm, resistance development was more frequent in subjects with baseline HIV-1 RNA >100,000 copies/mL compared to baseline HIV-1 RNA ≤100,000 copies/mL (9.0% *vs.* 3.5%, respectively) [14]. These results are consistent with the current indication for RPV/FTC/TDF in treatment-naïve patients, which is restricted to use in patients with HIV-1 RNA ≤100,000 copies/mL due to an increased risk of virologic failure and resistance development in patients with high baseline viral load [15,16].

To investigate the possibility that low-frequency mutant viruses were present at baseline in subjects that experienced virologic failure with resistance development in the STaR study, deep sequencing was performed on baseline samples from these subjects compared to control subjects that were virologic responders. Virologic failure samples from subjects that developed resistance were also analyzed to further characterize the resistance patterns that emerged.

## 2. Materials and Methods

### 2.1. STaR Study Design

Details of the STaR study have been previously published [12,13]. Briefly, STaR (GS-US-264-0110; clinicaltrials.gov identifier: NCT01309243) was a phase 3b, randomized, open-label, multi-center, international, 96-week study evaluating the safety and efficacy of the RPV/FTC/TDF STR compared to the EFV/FTC/TDF STR in treatment-naïve HIV-1 infected subjects. Subjects were randomized 1:1 to RPV/FTC/TDF or EFV/FTC/TDF. Eligibility criteria included screening HIV-1 RNA ≥2500 copies/mL, no prior ARV therapy, genotypic sensitivity to EFV, FTC, and TDF, and lack of the RPV mutations K101E/P, E138A/G/K/Q/R, Y181C/I/V, and H221Y by population sequencing. Randomization was stratified by screening HIV-1 RNA (≤100,000 or >100,000 copies/mL).

### 2.2. FDA Snapshot Outcome

The primary endpoint of the STaR study was the proportion of subjects with HIV-1 RNA <50 copies/ml at Week 48 by the FDA snapshot algorithm [12]. Efficacy by FDA snapshot algorithm at Week 96 (end of study) was a secondary objective of the study [13]. According to the FDA snapshot algorithm, results were classified as virologic success if the subject’s HIV-1 RNA was <50 copies/mL within the visit window. Results were classified as virologic failure if a subject’s HIV-1 RNA was ≥50 copies/mL, the subject had discontinued study drug due to lack of efficacy per the investigator’s assessment, or the subject discontinued due to reasons other than adverse event or death and the last on-study drug HIV-1 RNA was ≥50 copies/mL. Results were classified as no data in the visit window if a subject discontinued due to adverse event or death prior to the visit window, the subject discontinued due to reasons other than adverse event or death and the last on-study drug HIV-1 RNA was <50 copies/mL, or the subject was on study but had missing data in the visit window [17].

### 2.3. Resistance Analysis Population (RAP)

The resistance analysis population (RAP) included all subjects who qualified for resistance analysis through Week 96 of the STaR study. Subjects with suboptimal virologic response or virologic rebound (as defined below) and HIV-1 RNA ≥400 copies/mL who were on study drugs were considered to have virologic failure and were included in the RAP. Suboptimal virologic response was assessed at Week 8 and was defined as having HIV-1 RNA ≥50 copies/mL and <1 log_10_ reduction from baseline at the Week 8 visit, which was confirmed at the subsequent visit. Virologic rebound was defined as having two consecutive visits with HIV-1 RNA ≥50 copies/mL after achieving HIV-1 RNA <50 copies/mL, or as having two consecutive visits with >1 log_10_ increase in HIV-1 RNA from nadir. The sample from the confirmation visit (if available) was analyzed for resistance development. In addition, subjects who were on study drugs, had not been analyzed previously, and had HIV-1 RNA ≥400 copies/mL at Week 48, Week 96, or their last visit (at or after Week 8) were also included in the RAP. Population sequencing results for subjects in the RAP have been previously reported in detail [14,18].

### 2.4. Deep Sequencing Sample Selection

A total of 184 samples were analyzed using deep sequencing. These included 33 baseline and 33 virologic failure time point samples from subjects that met the criteria for inclusion in the RAP through Week 96 (24 RPV/FTC/TDF, 9 EFV/FTC/TDF). In addition, 118 baseline samples from control subjects with virologic response that did not qualify for the RAP (44 RPV/FTC/TDF, 74 EFV/FTC/TDF) were also analyzed. Non-RAP control subjects were chosen as a representative population matched with RAP subjects based on baseline HIV-1 RNA and CD4 cell count.

### 2.5. Virology Assessments

#### 2.5.1. Deep Sequencing Assay

Genotypic resistance testing was performed using the “deepTypeHIV” assay (SeqIT GmbH & Co. KG, Kaiserslautern, Germany). Viral RNA was isolated from patient plasma using QIAamp viral RNA mini kit (Qiagen, Hilden, Germany) according to the manufacturer’s protocol. Reverse transcription and polymerase chain reaction (PCR) were carried out using SuperScript III One-Step RT-PCR System with Platinum^®^ Taq High Fidelity (Life Technologies, Darmstadt, Germany) and primers 1RES, 5′-GAAGAAATGATGACAGCATGTCAGGG-3′ (nt 1819–1844, HXB2 numbering) and 2RES, 5′-TAATTTATCTACTTGTTCATTTCCTCCAAT-3′ (nt 4173–4202). Nested PCR was carried out with Platinum^®^ Taq High Fidelity (Life technologies) and the following inner primer pair: RES3, 5′-AGACAGGCTAATTTTTTAGGGA-3′ (nt 2074–2095) and RES4, 5′-ATGGYTCTTGATAAATTTGATATGTCC-3′ (nt 3559–3585). All amplicons were purified using the Agencourt AMPure^®^XP system on a BioMek NX workstation (Beckman Coulter, Krefeld, Germany), quantified fluorometric on a FluoStar Optima (BMG Labtech, Ortenberg, Germany) using Quant-iT Picogreen dsDNA reagent (Life Technologies).

Library preparation for Illumina deep sequencing was done using a Nextera^®^ XT DNA Sample Preparation and Index kit (Illumina, San Diego, CA, USA) according to the manufacturer’s manual. Resulting libraries were normalized and pooled for subsequent sequencing on an Illumina MiSeq platform using the 2 × 250 cycle paired-end sequencing protocol.

The clinical cutoff for the “deepTypeHIV” assay is 2% based on the background signal present at mutational positions of interest [19]. The 2% cutoff for the “deepTypeHIV” assay was confirmed by sequencing mixtures of HIV-1 xxLAI containing the M184V and K65R mutations in RT with wild-type HIV-1 xxLAI at 10%, 5%, 2%, 1%, 0.5%, and 0.2% mutant frequencies.

#### 2.5.2. Deep Sequencing Analysis

Analysis of deep sequencing data was performed using an internally-developed analysis pipeline where sequence reads in the form of FASTQ files were processed and aligned via a multi-step method.

To align the deep sequencing (next generation sequencing; NGS) results, a *de novo* assembly sequence from the baseline sample was used as the alignment sequence. The assembly sequence was created by the following steps: (1) contigs from paired-end reads were assembled using VICUNA [20]; and (2) contigs were then merged to generate an assembly sequence based on the alignment to the HIV reference sequence HIV-1 NL4-3 (Genbank Accession AF324493). Insertions and deletions (indels) were included in the assembly sequence for which the indel(s) did not result in a frameshift and were present in >50% of the viral population. Nucleotide mixtures were included in the assembly sequence when present at >15% of the viral population.

After a subject assembly sequence was created, the sequence reads were evaluated based on quality scores and aligned to the assembly sequence using MOSAIK v1.1.0017 [21]. More specifically, sequence reads were trimmed and filtered based on quality score and length. Trimming was performed for any reads with quality score <15, and any reads <50 nucleotides in length were excluded after trimming. All aligned reads were translated in-frame and changes from the reference sequence (HIV-1 NL4-3) were tabulated.

Mutations occurring at ≥2% were reported. Average coverage across primary nucleoside reverse transcriptase inhibitor (NRTI)- and NNRTI-associated resistance mutations was 18,802 reads per position for all samples tested.

#### 2.5.3. Population Sequencing Assay

At screening, pre-existing resistance in the protease (PR) and reverse transcriptase (RT) portion of the pol gene was assessed by genotype (population sequencing) using the GeneSeq™ assay (Monogram Biosciences, San Francisco, CA, USA). These screening data were used for baseline resistance analyses. For post-baseline resistance analyses of subjects in the RAP, PR/RT genotyping (population sequencing) and phenotyping were performed using the PhenoSense GT™ assay (Monogram Biosciences) [22].

#### 2.5.4. Viral Load Assay

Viral load (HIV-1 RNA copies/mL) was assessed from plasma using COBAS AMPLICOR Monitor Version 1.5 (Roche Diagnostics, Basel, Switzerland).

#### 2.5.5. Mutant Copy Number

Mutant copy number at baseline was calculated by multiplying the deep sequencing variant frequency (%) by the viral load (HIV-1 RNA copies/mL).

## 3. Results

Baseline characteristics of the subjects whose samples were analyzed by deep sequencing are described in Table 1. Baseline characteristics were generally similar between RAP and non-RAP control subjects as well as to the overall study population. The proportion of subjects with baseline HIV-1 RNA >100,000 copies/mL included in the deep sequencing analysis was greater than the proportion enrolled in the overall study population (56% *vs.* 35%, respectively) [12,13].

Primary NRTI and NNRTI drug resistance mutations detected by deep sequencing at low frequency (≥2% to 20%) in baseline samples and the list of mutations assessed are presented in Table 2. No drug resistance mutations were detected at low frequency in baseline samples from RAP subjects while primary NRTI or NNRTI drug resistance mutations were detected at low frequency in 8.5% of samples from control subjects (6.6% of all baseline samples tested). No subject had both primary NRTI and NNRTI mutations present at low frequency at baseline by deep sequencing. Using a more extensive list of mutations including the NNRTI mutations V90I, A98G, K101H, V106I, and V179D/F/I/T, and the NRTI mutations E44D, A62V, T69D, V75I, F77L, F116Y, and V118I, the prevalence of pre-existing low-frequency mutations at baseline was 15.2% overall.

**Table 1:** Baseline characteristics of subjects included in deep sequencing analysis.

Characteristic	RPV/FTC/TDF RAP *N* = 24	EFV/FTC/TDF RAP *N* = 9	Non-RAP Controls *N* = 118
Median age, years (IQR)	37 (31, 45)	28 (22, 33)	40 (31, 47)
Male, %	96%	89%	91%
White race, %	50%	44%	62%
Mean baseline CD4 cell count, cells/mm^3^ (SD)	253 (199)	370 (175)	329 (206)
Mean baseline HIV-1 RNA, log_10_ copies/mL (SD)	5.9 (6.2)	5.4 (5.7)	5.7 (6.1)
≤100,000 copies/mL, n (%)	11 (46%)	5 (56%)	52 (44%)
>100,000 copies/mL, n (%)	13 (54%)	4 (44%)	66 (56%)
HIV-1 Subtype, n (%)			
A	0	0	1 (0.85%)
AE	0	0	3 (2.54%)
AG	0	0	1 (0.85%)
B	23 (95.8%)	8 (88.9%)	102 (86.4%)
C	0	0	3 (2.54%)
D	0	0	1 (0.85%)
G	0	0	2 (1.70%)
Complex	1 (4.2%)	1 (11.1%)	5 (2.54%)

**Table 2:** Number of subjects with primary NRTI and NNRTI drug resistance mutations detected at low frequency (≥2% to 20%) in baseline samples by deep sequencing.

	RPV/FTC/TDF RAP *N* = 24	EFV/FTC/TDF RAP *N* = 9	Non-RAP Controls *N* = 118	Total *N* = 151
No mutations, n (%)	24 (100%)	9 (100%)	108 (91.5%)	141 (93.4%)
Any NRTI-R ^a^ or NNRTI-R ^b^ n (%)	0	0	10 (8.5%)	10 (6.6%)
Any NRTI-R, n (%)	0	0	3 (2.5%)	3 (2.0%)
Any NNRTI-R, n (%)	0	0	7 (5.9%)	7 (4.6%)

^a^ Primary nucleoside/nucleotide reverse transcriptase inhibitor resistance (NRTI-R) mutations are M41L, K65N/R, D67N, T69 insertion/deletion, K70E/R, L74V/I, Y115F, Q151M, M184I/V, L210W, T215Y/F, and K219E/Q/N/R in RT; ^b^ Primary non-nucleoside reverse transcriptase inhibitor resistance (NNRTI-R) mutations are L100I, K101E/P, K103N/S, V106A/M, V108I, E138A/G/K/Q/R, V179L, Y181C/I/V, Y188C/H/L, G190A/E/Q/S, H221Y, P225H, F227C, and M230I/L in RT.

Most non-RAP control subjects (eight out of 10) with primary NRTI and NNRTI drug resistance mutations present at low frequency at baseline were virologic successes (HIV-1 RNA <50 copies/mL) by the FDA snapshot algorithm at Week 96 (Table 3). The two subjects without virologic success in the EFV/FTC/TDF arm with low-frequency NRTI resistance mutations were lost to follow up after an initial virologic response.

In addition to minority species, primary NRTI or NNRTI drug resistance mutations present at levels >20% by deep sequencing were detected in baseline samples from four non-RAP control subjects and one RAP subject. None of the mutations detected were study protocol exclusion mutations. In the non-RAP control group, one subject in the EFV/FTC/TDF arm had D67N and K219Q detected at >99% frequency by deep sequencing. These same mutations were detected by population sequencing at screening and the subject was a virologic success at Week 96. Three additional control subjects had V108I detected at baseline. Two of these subjects (V108I at 30.4% and 98.8% by deep sequencing) also had V108I detected by population sequencing at screening and were virologic successes at Week 96 (one in each treatment arm). The third subject had V108I at 79.6% by deep sequencing at baseline that was not detected by population sequencing at screening. This subject was in the EFV/FTC/TDF arm and was dosed, but withdrew consent after a single day on study and therefore had no data in the Week 96 visit window. One RPV/FTC/TDF RAP subject had K219R at 99% by deep sequencing at baseline; this subject also had K219R present by population sequencing at screening, baseline, and virologic failure.

**Table 3:** Primary NRTI and NNRTI drug resistance mutations detected at low frequency (≥2% to 20%) in baseline samples from control subjects by deep sequencing and Week 96 snapshot outcome.

Subject Number ^a^	Baseline HIV-1 RNA (Copies/mL)	NRTI-R ^b^ (% Frequency)	NNRTI-R ^c^ (% Frequency)	Mutant Copy Number	Week 96 Snapshot Outcome
**RPV/FTC/TDF**					
1	3,620,000	K65R (2.00)	--	72,400	Success
2	562,000	--	V108I (2.23)	12,533	Success
3	535,000	--	E138K (2.11)	11,289	Success
4	1,240,000	--	Y181C (2.87)	35,588	Success
5	92,200	--	G190E (3.73)	3,439	Success
6	527,000	--	M230I (5.31)	27,984	Success
**EFV/FTC/TDF**					
7	61,400	M41L (12.3)	--	7,552	No data in window ^d^
8	2,500,000	K219Q (2.36)	--	59,000	Failure ^e^
9	164,000	--	L100I (14.6)	23,944	Success
10	93,000	--	G190E (2.33)	2,167	Success

--, no mutations. ^a^ All subjects had HIV-1 subtype B; ^b^ Primary nucleoside/nucleotide reverse transcriptase inhibitor resistance (NRTI-R) mutations are M41L, K65N/R, D67N, T69 insertion/deletion, K70E/R, L74V/I, Y115F, Q151M, M184I/V, L210W, T215Y/F, and K219E/Q/N/R in RT; ^c^ Primary non-nucleoside reverse transcriptase inhibitor resistance (NNRTI-R) mutations are L100I, K101E/P, K103N/S, V106A/M, V108I, E138A/G/K/Q/R, V179L, Y181C/I/V, Y188C/H/L, G190A/E/Q/S, H221Y, P225H, F227C, and M230I/L in RT; ^d^ Subject was lost to follow-up. HIV-1 RNA at last visit on study (Week 84) was <50 copies/mL; ^e^ Subject was lost to follow-up. HIV-1 RNA at last visit on study (Week 16) was 207 copies/mL.

Deep sequencing of samples from the failure time point in RAP subjects was generally consistent with the population sequencing results from the same time point (Table 4). Deep sequencing detected additional primary NRTI and NNRTI drug resistance mutations that were not detected by population sequencing in five RPV/FTC/TDF RAP subjects and two EFV/FTC/TDF RAP subjects. Most of these mutations were present at low frequency (≥2% to 20%) and also contained mutations of the same drug class present at levels >20% that were detected by population sequencing.

**Table 4:** Primary NRTI and NNRTI drug resistance mutations detected in virologic failure samples from RAP subjects by deep sequencing compared with population sequencing.

Subject Number	Baseline HIV-1 RNA (copies/mL)	HIV-1 RNA at VF (copies/mL)	Deep Sequencing		Population Sequencing	
			NRTI-R ^a^ (% Frequency)	NNRTI-R ^b^ (% Frequency)	NRTI-R ^a^	NNRTI-R ^b^
**RPV/FTC/TDF**						
1	2,330,000	586,000	M184V (99)	K101E (64) **E138K (11)** Y181I (85)	M184V	K101K/E Y181I
2	2,140,000	824,000	**D67N (2.4)** M184I (>99)	E138K (96) **E138Q (4)** H221Y (49)	M184I	E138K H221H/Y
3	98,900	9,370	**D67N (62)** M184V (35)	--	*K65K/R* M184M/I/V	--
4	63,000	343,000	M184V (>99) **K219E (5.7)** **K219N (5.6)**	Y181C (>99)	M184V	Y181C
5	12,400	70,600	M184I (>99) K219R (>99)	E138K (34) E138Q (66) **H221Y (34)**	M184I K219R ^d^	E138K/Q
6	6,410,000	1,530,000	M184I (>99)	K101E (58) E138K (45) Y181C (44) H221Y (42)	M184I	K101K/E E138E/K Y181Y/C H221H/Y
7	2,510,000	20,500	K65R (>99) T69del (24) K219E (>99)	Y181C (99) Y188H (99)	K65R T69T/del K219K/E	Y181C Y188H
8	891,000	46,600	M184V (>99)	K101E (18) Y181I (>99)	M184V	K101K/E Y181I
9	621,000	409,000	M184V (>99)	E138K (>99) Y181I (>99)	M184V	E138K Y181I
10	564,000	24,000	M184I (>99)	E138Q (>99)	M184I	E138K/Q
11	288,000	36,500	Y115F (>99) M184I (99) K219E (69)	K101E (99)	Y115Y/F M184I K219K/E	K101E *Y181Y/C*
12	215,000	24,100	M184I (>99)	E138K (>99) H221Y (6.1)	M184I	E138K H221H/Y
13	127,000	1,120	K70E (>99) M184I (>99)	E138K (>99)	K70K/E M184I	E138K
14	65,100	489	M184V (64)	V108I (61) F227C (62)	*K65K/N* M184M/I/V	V108V/I F227F/C *M230M/I*
15	314,000	998	M184I (>99)	--	M184I	--
16	59,100	2,750	M184I (>99)	E138K (>99)	M184I	E138K
17	50,700	798	M184I (>99)	E138K (>99) H221Y (40)	M184I	E138K H221H/Y
18	50,600	2,570	L74V (>99) M184V (>99)	L100I (>99) K103N (99) P225H (99)	L74V M184V	L100I K103N P225H
19	44,400	7,590	M184I (>99) K219E (>99)	K101E (43) Y181C (>99) M230L (>99)	M184I K219E	K101E Y181C M230L
20	10,500	1,980	M184I (>99)	E138K (>99)	M184I	E138K
21	522,000	527	AF	AF	--	--
22	60,600	518	AF	AF	--	--
23	154,000	659	--	--	--	--
24	20,600	24,700	--	--	--	--
**EFV/FTC/TDF**						
1	1,440,000	192,000	**K65R (7.8)** D67N (99) M184I (>99) K219E (50) **K219N (2.4)**	G190E (55) G190Q (44) **H221Y (8.7)**	D67N M184I K219K/E	G190E/Q
2	150,000	485	--	**M230L (>99)**	--	--
3	97,700	648	M184I (>99)	M230L (>99)	M184M/I	M230M/L
4	32,400	24,700	--	K103N (99)	--	K103N
5	11,200	3,020	--	Y188L (>99)	--	Y188L
6	452,000	46,600	--	--	--	--
7	161,000	205,000	--	--	--	--
8	22,500	20,100	--	--	--	--
9	22,100	2,020	--	--	--	--

--, no mutations. AF, assay failure; VF, virologic failure. Mutations detected by deep sequencing but absent by population sequencing are underlined and bolded. Mutations detected by population sequencing but absent by deep sequencing are underlined and italicized. ^a^ Primary nucleoside/nucleotide reverse transcriptase inhibitor resistance (NRTI-R) mutations are M41L, K65N/R, D67N, T69 insertion/deletion, K70E/R, L74V/I, Y115F, Q151M, M184I/V, L210W, T215Y/F, and K219E/Q/N/R in RT; ^b^ Primary non-nucleoside reverse transcriptase inhibitor resistance (NNRTI-R) mutations are L100I, K101E/P, K103N/S, V106A/M, V108I, E138A/G/K/Q/R, V179L, Y181C/I/V, Y188C/H/L, G190A/E/Q/S, H221Y, P225H, F227C, and M230I/L in RT; ^c^ Subject had a complex mixture of HIV-1 subtypes. All other subjects had HIV-1 subtype B; ^d^ Subject also had K219R present by population sequencing at the screening and baseline visits.

Three samples with mutant frequencies of >20% had discrepant results for deep sequencing and population sequencing at the virologic failure time point. In two cases, the discrepancies occurred in subjects with HIV-1 RNA <500 copies/mL (less than the validated limit of detection for both assays) in the virologic failure sample. EFV/FTC/TDF RAP subject #2 had HIV-1 RNA 485 copies/mL with M230L detected by deep sequencing at >99% frequency, but had no mutations detected by population sequencing. RPV/FTC/TDF RAP subject #14 had HIV-1 RNA 489 copies/mL with M184V (63%) and F227C (62%) detected by deep sequencing and K65K/N, M184M/I/V, V108V/I, F227F/C, and M230M/I present by population sequencing. RPV/FTC/TDF RAP subject #3 had HIV-1 RNA 9370 copies/mL with D67N (62%) and M184V (35%) detected by deep sequencing and K65K/R and M184M/I/V present by population sequencing.

## 4. Discussion

Baseline HIV-1 primary NNRTI- and NRTI-resistance associated mutations were detected as minority populations (frequencies >2% and ≤20%) in 6.6% of subject isolates analyzed by deep sequencing in the STaR study. The prevalence of low-frequency drug resistance species in the current study is slightly lower than that observed in other studies of treatment-naïve patients [3,4,10,23]. This difference may be due in part to the use of a more focused mutation list of primary NNRTI- and NRTI-resistance mutations known to have significant impact on susceptibility to the components of RPV/FTC/TDF and EFV/FTC/TDF, and did not include minor or polymorphic mutations that may have been part of mutation lists used in other studies.

No RPV/FTC/TDF or EFV/FTC/TDF-treated subjects who experienced virologic failure with resistance development had pre-existing primary NNRTI- or NRTI-resistant virus populations at levels >2% and ≤20%. While the presence of pre-existing low-frequency resistance mutations does not appear to explain the higher rate of virologic failure in high baseline viral load subjects treated with RPV/FTC/TDF observed in the STaR study, it is possible that subjects with high baseline viral load have greater viral diversity, including more virus species containing drug resistance mutations potentially present below the limit of detection of the assay used in this study, and this could impact clinical outcome. The majority of subjects (seven out of 10) who had low-frequency primary NNRTI- or NRTI-resistance mutations present at baseline also had HIV-1 RNA >100,000 copies/mL, supporting the idea that subjects with high baseline viral load have greater variation in their virus population that pre-exists as a result of errors generated during viral replication driven by HIV-1 reverse transcriptase. However, the high virologic response rates among the few subjects with low-frequency pre-existing resistance in the STaR study suggest that other factors such as adherence, CD4 cell count, or drug exposure are playing a more significant role in determining virologic outcome in high baseline viral load subjects.

All subjects with low-frequency populations of primary NNRTI- or NRTI-associated resistance mutations present at baseline in the current study had single mutations detected, which may not have been sufficient to influence virologic response. It is possible that the presence of multiple mutations and/or mutations to multiple classes of antiretroviral drugs may be needed in order for low-level mutations to increase the risk of virologic failure. However, a recent study in treatment-experienced patients found that neither single nor dual-class minority resistant viruses at baseline had a significant impact on virologic response [7]. It is also possible that particular mutations or classes of mutations may have a greater impact on clinical outcomes than others. Furthermore, our study was limited by the exclusion of subjects with NNRTI or NRTI resistance mutations present by population sequencing at screening, the potential exclusion of additional subjects with pre-existing low-frequency resistance mutations who did not meet other enrollment criteria, the limited number of subjects that developed resistance during the study, and the observation that most low-frequency mutations detected at baseline were very close to the deep sequencing assay cutoff of 2%.

Previous studies have suggested a dose-dependent relationship between the absolute copy number of a particular viral mutant (or mutational load) and impact on virologic outcome [8,24]. This is thought to be particularly relevant for the NNRTI mutation K103N which has been associated with an increased risk of virologic failure on EFV-based regimens if present above a threshold of 2000 copies/mL [24]. Mutant copy number did not appear to play a significant role in the current study since most of the subjects with a pre-existing low-frequency mutant had HIV-1 RNA >100,000 copies/mL, resulting in a mutant copy number of greater than 2000 copies/mL in all cases as well as a high virologic success rate.

Deep sequencing results were concordant with the population sequencing results in the majority of samples. Additional low-frequency primary NRTI and NNRTI drug resistance mutations were detected by deep sequencing in five virologic failure samples in which mutations of the same class had also been detected by population sequencing. In addition, there were a small number of instances (one baseline sample and three virologic failure samples) where the assays produced discrepant results for mutations with frequencies >20%. Since the deep sequencing and population sequencing assays are from different clinical laboratories and use different sets of primers for the reverse transcription and PCR steps of the assays, it is possible that each assay amplified a slightly different virus population due to differences in primer sensitivity and/or specificity. Furthermore, two of the virologic failure samples with discrepancies had HIV-1 RNA <500 copies/mL, which is below the validated limit of detection for both assays. The limited virus present in these samples may have led to increased differential amplification between the two assays and potential over- or underestimation of the variants present. All of the mutations detected by population sequencing that were not detected by deep sequencing were present as mixtures with wild-type and may have been due to the overcalling of trace signals by population sequencing.

Numerous studies have examined the effect of minority populations of drug resistance-associated virus to impact treatment outcome in treatment-naïve patients, particularly for NNRTI-based regimens. Some studies have reported an increased risk of virologic failure associated with the presence of pre-existing low-frequency drug resistant variants [2,8,9,10]. Others have found limited to no evidence for a role of low-frequency variants in virologic response, consistent with the results of the current study [3,5,6,11]. Notably, our results are in agreement with the deep sequencing analysis from subjects who failed treatment with the components of RPV/FTC/TDF in the pivotal ECHO and THRIVE studies, demonstrating that the mutations that emerged in subjects on RPV-containing regimens were not present as low-frequency variants at baseline [25]. It is possible that variants that are transmitted and present at low frequency prior to treatment initiation may have a different impact on treatment outcomes than low-frequency drug resistance virus species arising under selective drug pressure in treatment-experienced patients. The clinical significance of minority drug resistant variants requires further investigation, particularly with regard to different treatment regimens and patient populations.

Deep sequencing of HIV-1 patient isolates is currently still an experimental technique that is not routinely used in clinical practice to inform patient management as the clinical significance of low-frequency drug resistant variants remains unclear. Since deep sequencing can be cost-effective compared to population sequencing, it is possible that deep sequencing may replace population sequencing as standard of care in the future. In this case, prospective clinical data should be utilized to determine the clinical significance of variant frequencies or mutational burden by position and specific antiretroviral regimen.
